# Transcriptomic and proteomic analyses of the immune responses of C-type lectin from *Conogethes punctiferalis* against fungal infection

**DOI:** 10.3389/fimmu.2026.1774776

**Published:** 2026-02-26

**Authors:** Shaohua Li, Zhiwei Kang, Xiangdong Li, Hailei Wei, Xiangchu Yin, Fanghua Liu, Fangqiang Zheng

**Affiliations:** 1 College of Plant Protection, Shandong Agricultural University, Tai’an, Shandong, China; 2 College of Resources and Environment, Shandong Agricultural University, Tai’an, Shandong, China; 3 College of Life Science, Hebei University, Baoding, Hebei, China; 4 Institute of Plant Protection, Shandong Academy of Agricultural Sciences, Jinan, Shandong, China; 5 Institute of Agricultural Resources and Regional Planning, Chinese Academy of Agricultural Sciences, Beijing, China

**Keywords:** *Beauveria bassiana*, *Conogethes punctiferalis*, C-type lectin, insect immunity, proteomic, transcriptomic

## Abstract

**Introduction:**

C-type lectins (CTLs), a family of pattern recognition receptors, participate in insect innate immunity and could serve as potential targets for insect pest management. However, information about CTLs in the immune responses of *Conogethes punctiferalis*, a destructive insect pest damaging to maize in China, has received minimal attention.

**Methods:**

The integrative transcriptomic and proteomic analyses of non-infected and *Beauveria bassiana*-infected *C*. *punctiferalis* larvae were performed using RNA-Seq and iTRAQ techniques. The survival rates and phenotypic changes of larvae infected with *B*. *bassiana* were investigated using RNA interference (RNAi).

**Results:**

Based on the transcriptome and proteome data, screening identified 314 immune-related genes and proteins, including 14 CTLs. According to the number and organization of carbohydrate recognition domains (CRDs), six CTLs were classified as CTL-S (single CRD), while eight CTLs were classified as IML (dual CRD). The expression of a novel CTL, designated *CpIML4*, increased in response to *B*. *bassiana* infection. The developmental stage and larval tissue expression analyses showed that *CpIML4* was highly expressed in the 5^th^-instar larvae and their hemolymph, respectively. RNAi-mediated knockdown of *CpIML4* significantly decreased resistance to *B. bassiana*, as indicated by lower survival rates and pathological phenotypic changes of larval cuticle. The larvae exhibited developmental malformation and black spots on the cuticle, and the dead larvae turned black and wrinkled.

**Discussion:**

These results demonstrate that *CpIML4* might play a crucial role in the immune responses of *C*. *punctiferalis* against *B*. *bassiana* infection. Collectively, our findings not only provide a comprehensive view of the immune responses of *C*. *punctiferalis* to *B. bassiana* infection, but also enhance our understanding of *C*. *punctiferalis* CTLs and highlight candidate genes for RNAi-mediated insect pest control.

## 1 Introduction

Unlike vertebrates, insects lack lymphocyte-mediated adaptive immune systems; nonetheless, they are capable of adapting to adverse environments rich in various pathogenic microorganisms (1). This adaptation is attributed to their powerful and highly-developed innate immune systems, which include cellular and humoral immunity (2). To combat infections by various pathogenic microorganisms, insects have evolved highly sensitive recognition mechanisms and effective immune defense strategies (2). Pattern recognition receptors (PRRs) are proteins encoded by the germline, which are able to recognize and bind to pathogen-associated molecular patterns (PAMPs) to induce a cascade of downstream immune responses (3). The most common PRRs in insects include peptidoglycan recognition proteins (PGRPs), Gram-negative bacteria binding proteins (GNBPs), and C-type lectins (CTLs) (4). Among them, CTLs are a large superfamily of proteins that exist in insects as well as other invertebrates, vertebrates, and even plants (5, 6).

CTLs comprise calcium ion (Ca^2+^)-dependent carbohydrate-binding proteins that recognize a variety of glycoconjugates via carbohydrate recognition domains (CRDs), which are also known as C-type lectin-like domains (CTLDs) (7, 8). The typical CRD consists of 110 to 130 amino acid residues and has a double-loop structure containing two *α*-helices, antiparallel *β*-sheets, and two or three pairs of conserved disulfide bonds (9, 10). In insects, CRDs are essential for determining the ligand-binding specificity of CTLs, which depends on their tripeptide motifs. For example, EPN (Glu-Pro-Asn) exhibits a higher binding affinity for mannose-type ligands, whereas QPD (Gln-Pro-Asp) demonstrates a stronger binding affinity for galactose-type ligands (9, 11). Atypical motifs have also been characterized in insects, and these include EPD (Glu-Pro-Asp), QPN (Gln-Pro-Asn), EPS (Glu-Pro-Ser), and QPR (Gln-Pro-Arg) (12, 13). Insect CTLs are classified into three categories based on CRD organization. CTLs with a single CRD (CTL-S) are common in most insects (14). Some CTLs with a dual CRD, namely immulectin (IML), exist widely in lepidopteran insects; for example, MsIML in *Manduca sexta* and BmLBP (lipopolysaccharide-binding protein), BmMBP (multi-binding protein), and BmLEL (low-expression lectin) in *Bombyx mori* (11, 14). Additionally, some CTLs that contain a single CRD with other functional domains have been identified in insects. These CTLs are also known as CTL-X and include complement control protein (CCP), complement C1r/C1s, Uegf, Bmp1 (CUB), epidermal growth factor-like domains (EGF), and immunoglobulin (IG) (12–14).

RNA sequencing (RNA-Seq) and isobaric tags for relative and absolute quantification (iTRAQ) techniques have been widely employed in entomological studies in recent years (15, 16). CTLs in some lepidopteran insects have been determined based on these analytical techniques (10–13, 17). The widely known functions of insect CTLs influence their innate immune responses, including prophenoloxidase (PPO) activation, melanization, opsonization, antimicrobial peptide (AMP) expression, hemocyte-mediated phagocytosis, nodulation, and encapsulation (8, 14, 18). For instance, *MsIML2* can stimulate phenol oxidase activation in the hemolymph and participate in the encapsulation and melanization in *M. sexta* (19, 20). In *B. mori*, CTLs can enhance the phagocytosis, nodulation, and encapsulation ability of hemocytes, regulate AMPs, PPOs, and apoptosis-related gene expressions, and even act as opsonins in the hemolymph to promote the elimination of pathogens (21–23).

The yellow peach moth, *Conogethes punctiferalis* (Lepidoptera: Crambidae), is a highly destructive agricultural insect pest with a wide distribution across subtropical and tropical Asia and Australia (24). As a polyphagous insect pest, it can damage more than 100 crop species, including field crops, fruit trees, and vegetables (25). In recent years, the population of *C*. *punctiferalis* has gradually increased on maize in the Huang-Huai-Hai region of China (26). Its larvae are able to tunnel into maize ears, thereby inducing fungal ear rot diseases and toxins (such as aflatoxin) production from those diseases, which not only results in serious economic losses to maize but also poses a threat to food safety (27). Currently, chemical insecticides are the most common and effective measure used to control *C*. *punctiferalis* (28). However, the extensive use of these products can result in environmental pollution and insecticide resistance; therefore, eco-friendly pest management agents are needed (29). Biological control using entomopathogenic fungi is an effective alternative strategy to chemical insecticides for insect pest control (30). *Beauveria bassiana*, a common entomopathogenic fungus, offers a promising and environmentally friendly alternative, and it has been widely applied in insect pest management (31). The innate immune system of insects is a key factor affecting the fungal biocontrol potential (32), and understanding this can improve the effectiveness of applying fungal biocontrol agents. Recently, RNAi has also emerged as a valuable technology in the management of species-specific insect pests (33). For example, the United States Environmental Protection Agency has approved a sprayable RNAi-based bioinsecticide, Calantha™, that targets the Colorado potato beetle, *Leptinotarsa decemlineata*, to protect potato plants (34). Unlike traditional chemical insecticides, RNAi-based bioinsecticides are novel and valuable alternatives due to their strong efficacy, high specificity, and safety for the environment and non-target organisms (35). Noticeably, RNAi-mediated insect pest control is largely determined by the selection of key target genes (36). CTLs are important immune recognition molecules in insects and may represent potential targets for insect pest control. However, knowledge on CTLs in *C*. *punctiferalis* during fungal infections remains poorly understood.

In this study, the innate immune responses of *C*. *punctiferalis* larvae following fungal infection were investigated by RNA-Seq and iTRAQ techniques. The CTLs of *C*. *punctiferalis* larvae were screened and identified based on the transcriptome and proteome data. Furthermore, the roles of a CTL, named *CpIML4*, were analyzed using RNA interference (RNAi) after injection with *B*. *bassiana*. Our results contribute to a better understanding of CTL functions in insect immunity, and provide a new theoretical basis for RNAi-based insect pest control.

## 2 Materials and methods

### 2.1 Insect rearing and fungus culturing

The initial *C*. *punctiferalis* population was collected from maize fields in Tai’an City, Shandong province, China. All *C*. *punctiferalis* larvae were reared on fresh maize grains and adult moths were fed with a 10% (*v*/*v*) honey solution in an artificial climate incubator set at 25 ± 1 °C, 70 ± 5% relative humidity, and a photoperiod 14:10 (L:D) h.

The cultivation of *B*. *bassiana* strain (ACCC30107), preparation of conidial suspension, and method of *C*. *punctiferalis* larvae infected with *B*. *bassiana* were performed in accordance with our previous study (37). Then, the samples of larvae were frozen in liquid nitrogen at 12 h post injection (hpi) based on previous findings for further use (38).

### 2.2 Transcriptome sequencing and analysis

The total RNA of non-infected and *B*. *bassiana*-infected larvae was extracted using TRNzol Universal Reagent (Tiangen, Beijing, China). Each control or treatment group included nine larvae, and each bioassay was conducted in triplicate. High-quality RNA samples were used to construct libraries that were sequenced on an Illumina NovaSeq 6000 platform at Beijing Novogene Co., Ltd., China. Quality control, *de novo* transcriptome assembly, and gene functional annotation were conducted as previously described (39). The differentially expressed genes (DEGs) were identified by DESeq2 software. The screening criteria of significantly differential genes were set as *p* adj < 0.05 and |log_2_(fold change, FC)| > 1. GO and KEGG pathway enrichment analyses of DEGs were performed.

### 2.3 Proteome sequencing and analysis

The *C*. *punctiferalis* larvae samples used for proteome sequencing were the same as those for transcriptome sequencing. Protein extraction, iTRAQ labeling, and LC-MS/MS analysis were performed as previously described (37). Proteome sequencing analysis was also conducted using the iTRAQ technique at Beijing Novogene Co., Ltd., China. The raw data were analyzed by the Proteome Discoverer 2.2 software. The results of protein quantitation were statistically analyzed by *t*-test and the proteins with significant differences between the treatment group and the control group were selected (the up-regulated expression: FC ≥ 1.2 and *p* ≤ 0.05; the down-regulated expression: FC ≤ 0.83 and *p* ≤ 0.05) and defined as differentially expressed proteins (DEPs). Functional annotation and enrichment analysis of DEPs were performed as previously described (37).

### 2.4 Identification and bioinformatics analysis of CTLs

All CTLs of *C*. *punctiferalis* were retrieved and identified based on the transcriptome and proteome data. The open reading frame (ORF) of the CTLs was obtained using ORF finder (https://www.ncbi.nlm.nih.gov/orffinder/). The theoretical molecular weight (MW) and isoelectric point (pI) of the mature protein were calculated using ExPASy (http://web.expasy.org/compute_pi/). Predictions of the signal peptide, conserved domains, and transmembrane regions were made using SignalP 5.0 (https://services.healthtech.dtu.dk/service.php?SignalP-5.0), SMART (http://smart.embl-heidelberg.de/), and TMHMM (https://services.healthtech.dtu.dk/services/TMHMM-2.0/), respectively. NetNGlyc 1.0 (https://services.healthtech.dtu.dk/services/NetNGlyc-1.0/) and NetOGlyc 4.0 (https://services.healthtech.dtu.dk/services/NetOGlyc-4.0/) were used to predict the N-glycosylation and O-glycosylation sites, respectively. A three-dimensional (3D) structure model was predicted using COFACTOR and COACH based on the I-TASSER server (https://zhanggroup.org/I-TASSER/) (40). The results of generated PDB files were visualized using the PyMOL Molecular Graphics System 2.6 software.

The amino acid sequences of the CTLs were retrieved and downloaded from GenBank. The CTLs from *B. mori*, *Helicoverpa armigera*, *Spodoptera litura*, *M. sexta*, *Galleria mellonella*, *Ostrinia furnacalis*, *Drosophila melanogaster*, *Anopheles gambiae*, *Aedes aegypti*, *Rhynchophorus ferrugineus*, *Sitophilus oryzae*, *Dendroctonus ponderosae*, *Rhopalosiphum maidis*, *Rhopalosiphum padi*, *Bemisia tabaci*, and *Macrobrachium rosenbergii* were selected for multiple sequence alignment and phylogenetic analysis. Multiple amino acid sequences were aligned using ClustalW (https://www.genome.jp/tools-bin/clustalw) and decorated with ESPript 3.0 (https://espript.ibcp.fr/ESPript/cgi-bin/ESPript.cgi). Phylogenetic trees were constructed using MEGA 11.0 software through the neighbor-joining method (bootstrap = 1,000 replications).

### 2.5 cDNA cloning and sequencing analysis of *CpIML4*

The total RNA of 5^th^-instar larvae (3-day-old) was extracted and reverse transcribed into cDNA according to the manufacturer’s instructions. The coding sequence (CDS) of *CpIML4* from the transcriptome data was employed to design CDS-specific primers (*IML4*-F and *IML4*-R) using Primer Premier 6 software (**Supplementary Table 1**). The sequence of *CpIML4* was amplified by PCR using above primers. The PCR products were purified and ligated into the pMD™ 18-T Vector (TaKaRa, Japan). The positive clones were selected by PCR and confirmed by sequencing at Beijing Liuhe BGI Co., Ltd., China.

### 2.6 Expression profile analysis of *CpIML4*

Samples of the developmental stages, including eggs (1-day-old, 300 eggs), 1^st^-instar larvae (1-day-old, 100 individuals), 2^nd^-instar larvae (1-day-old, 80 individuals), 3^rd^-instar larvae (1-day-old, 40 individuals), 4^th^-instar larvae (1-day-old, 20 individuals), 5^th^-instar larvae (1-day-old, 10 individuals), pupae (1-day-old, 10 individuals), and adults (1-day-old, 10 individuals) were collected. Samples of different tissues, including the head (20 individuals), midgut (20 individuals), fat body (20 individuals), hemolymph (20 individuals), and cuticle (20 individuals), were collected from 3-day-old 5^th^-instar larvae. Three biological replicates were analyzed for each sample. The methods of total RNA extraction and cDNA synthesis were the same as those described above. Specific primers (q*IML4*-F and q*IML4*-R) for *CpIML4* were designed using Primer Premier 6 software (**Supplementary Table 1**). The *C*. *punctiferalis* ribosomal protein 49 (RP49) was used as an internal control in all trials. A reaction system of 20 μL was prepared including 10 μL of SuperReal PreMix Plus (2×), 1 μL of cDNA, 1 μL of upstream and downstream primer, and 7 μL of RNase-free ddH_2_O. The relative expression level of *CpIML4* was analyzed by qRT-PCR using a Bio-Rad CFX96 Touch Real Time PCR Detection System (Bio-Rad, USA) with the following setting: 95 °C for 15 min, 40 cycles of 95 °C for 10 s, and 60 °C for 30 s. All samples were analyzed in triplicate and procedures were repeated thrice as independent biological replicates. The 2^−ΔΔCT^ method (41) was used to determine the relative expression levels of *CpIML4*.

### 2.7 RNAi of *CpIML4*

The double-stranded RNA (dsRNA) of *CpIML4*, ds*IML4*, was synthesized using the T7 RiboMAX™ Express RNAi System (Promega, USA). Green fluorescent proteins (GFP) were employed to generate the dsRNA (ds*GFP*).Specific primers for the target gene (ds*IML4*-F, ds*IML4*-R, ds*IML4*-T7F, and ds*IML4*-T7R) and for the GFP gene (ds*GFP*-F, ds*GFP*-R, ds*GFP*-T7F, and ds*GFP*-T7R) were designed using Primer Premier 6 software (**Supplementary Table 1**). The dsRNA Off-target Minimization Generator (dsOMG) (https://dsomg.sysu.edu.cn) (42) was used to select ds*IML4* fragments with a low risk of off-target effects. The integrity of dsRNA was checked by 1% agarose gel electrophoresis. The NanoDrop One spectrophotometer (Thermo Fisher Scientific, USA) was used to measure the concentration of dsRNA, which was subsequently diluted with nuclease-free water to a final concentration of 1 μg/μL. A total of 2 μg of ds*IML4* was injected into the hemocoel of 5^th^-instar larvae (3-day-old) using a microinjector (Hamilton, Switzerland). Larvae injected with the same volume of ds*GFP* were used as controls. The larvae from the treatment and control groups were collected at 12, 24, and 36 hpi. The relative expression levels of *CpIML4* were validated by qRT-PCR.

### 2.8 Survival assay after *CpIML4* RNAi

The 5^th^-instar larvae (3-day-old, 120 individuals) were divided into four groups to evaluate their survival rates after *CpIML4* RNAi: (I) ds*GFP*+PBS, 30 larvae were injected with 2 μL sterile PBS after injection with ds*GFP* (as control); (II) ds*IML4*+PBS, 30 larvae were injected with 2 μL sterile PBS after *CpIML4* RNAi; (III) ds*GFP*+Bb, 30 larvae were injected with 2 μL *B*. *bassiana* conidial suspension (5 × 10^4^ conidia/μL) after injection with ds*GFP* (as a control); (IV) ds*IML4*+Bb, 30 larvae were injected with 2 μL *B*. *bassiana* conidial suspensions (5 × 10^4^ conidia/μL) after *CpIML4* RNAi. Three independent replicates were used in each trial. All larvae were maintained in the above-mentioned normal rearing conditions. The survival rates and phenotypic changes of larvae were recorded every 12 h until pupation or death.

### 2.9 Statistical analysis

Statistical analysis was conducted using IBM SPSS Statistics 26 software and GraphPad Prism 9.0 software. The temporal and spatial expression data were examined through a one-way ANOVA; RNAi efficiency was determined through an unpaired *t*-test; and the Kaplan-Meier survival rates were performed using the log-rank (Mantel-Cox) test. All data were evaluated for mean ± standard error (SE), and significant differences between values were defined as *p* < 0.05.

## 3 Results

### 3.1 Transcriptomic analysis

A total of 132,834,380 raw reads were generated, and 127,808,299 clean reads were obtained after quality filtration (**Supplementary Table 2**). In total, 135,925,102 transcripts and 45,921,295 unigenes were obtained using the Trinity 2.5.1 software and the Corset 4.6 software (**Supplementary Table 3**). A total of 33,648 unigenes were annotated in seven databases, including NR, NT, KEGG, SwissProt, Pfam, GO, and KOG (**Supplementary Table 4**). Among these, 11,978 unigenes were annotated and grouped into three GO categories (**Supplementary Figure 1A**). 6,000 unigenes were annotated and distributed across 26 KOG categories (**Supplementary Figure 1B**). 7,080 unigenes were annotated and clustered into five KEGG metabolic categories (**Supplementary Figure 1C**). According to the homology analysis, the majority of unigenes mapped to *O*. *furnacalis* (8,359 unigenes), followed by *Chilo suppressalis* (1,851 unigenes), and *H*. *armigera* (754 unigenes) (**Supplementary Figure 2**).

**Figure 1:**
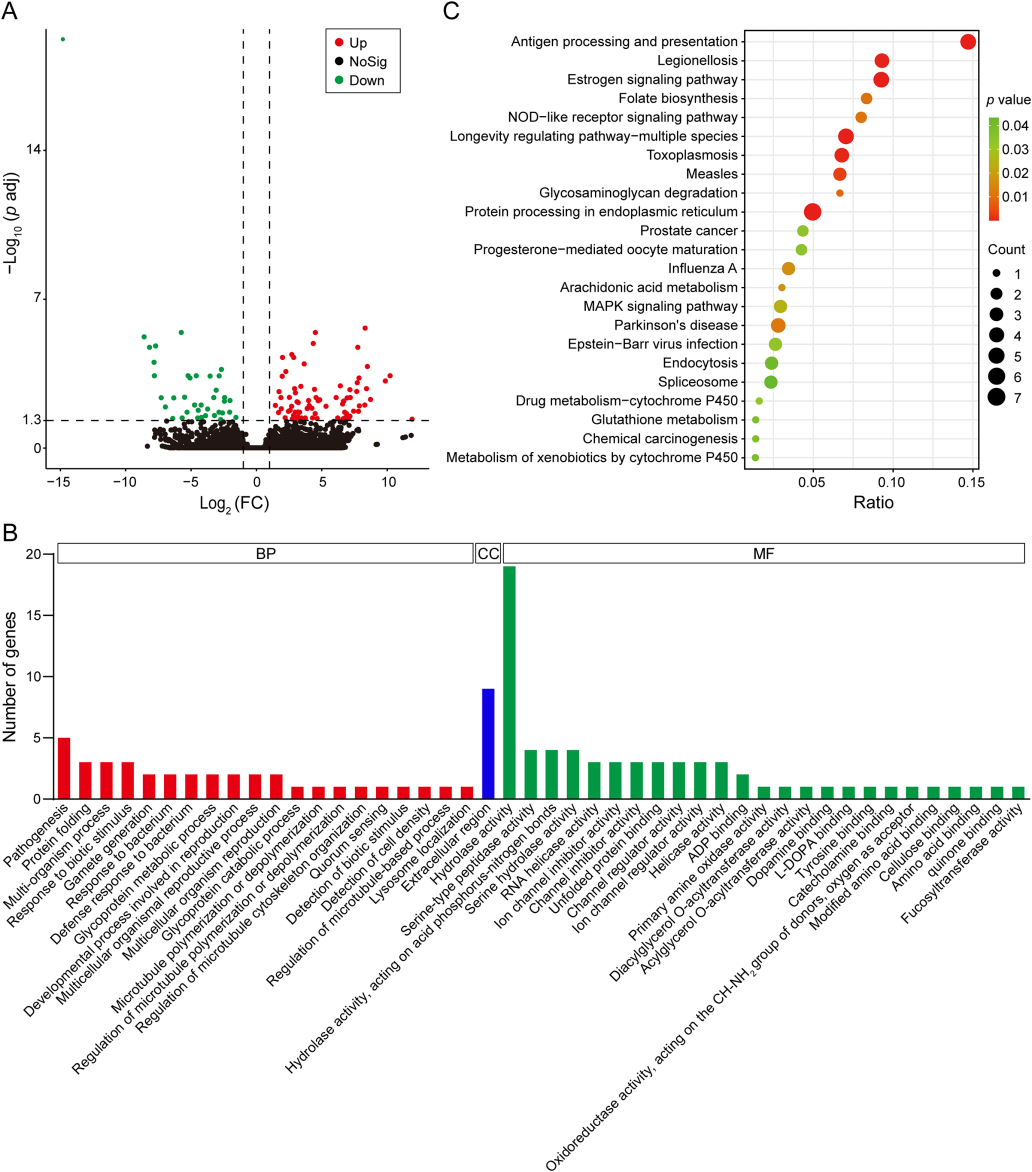
Transcriptome analysis of *C*. *punctiferalis* larvae infected with *B*. *bassiana*. **(A)** Volcano plot. **(B)** GO enrichment analysis of DEGs. **(C)** KEGG pathway enrichment analysis of DEGs.

According to the differential gene screening criteria, a total of 114 DEGs were identified, of which 76 were up-regulated and 38 were down-regulated (**Figure 1A**). A total of 54 DEGs were annotated to 452 GO terms based on GO enrichment analysis. Among these, the most enriched GO terms were “pathogenesis” (GO 0009405) in biological process, “extracellular region” (GO 0005576) in cellular component, and “hydrolase activity” (GO 0016787) in molecular function (**Figure 1B**). Based on the KEGG pathway enrichment analysis, 16 DEGs annotated to 34 KEGG terms. The most enriched KEGG pathways were “protein processing in endoplasmic reticulum” (ko 04141), “antigen processing and presentation” (ko 04612), “estrogen signaling pathway” (ko 04915), and “longevity regulating pathway-multiple species” (ko 04213) (**Figure 1C**).

### 3.2 Proteomic analysis

To improve the accuracy of the proteome data, protein quality controls were conducted (**Supplementary Figure 3**). In total, 67,130 spectra were matched to 583,724 total spectra, and 31,653 peptides, 3,544 identified proteins and 3,431 quantifiable proteins were checked and identified (**Supplementary Table 5**). In total, 1,468 proteins were functionally annotated in GO, COG, KEGG, and IPR databases (**Supplementary Figure 4**). Among these, a total of 2,336 proteins were annotated and grouped into three GO categories (**Supplementary Figure 5A**). A total of 1,832 proteins were annotated and distributed across 25 COG categories (**Supplementary Figure 5B**). A total of 3,399 proteins were annotated and clustered into five KEGG metabolic categories (**Supplementary Figure 5C**). A total of 3,088 proteins were annotated in the IPR analysis (**Supplementary Figure 5D**). Subcellular localization showed that 2,051 proteins were classified into 14 categories (**Supplementary Figure 5E**).

**Figure 2:**
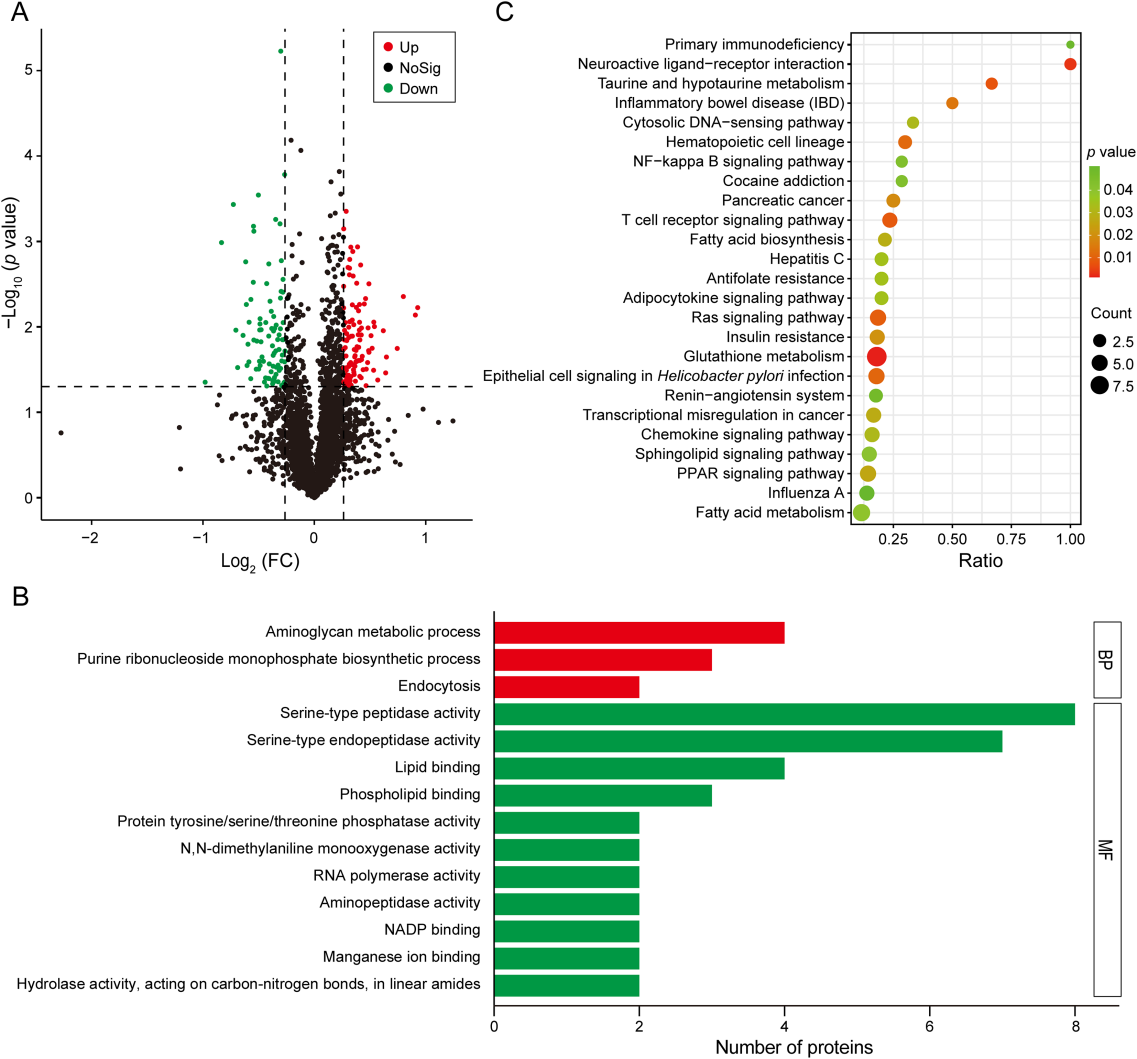
Proteome analysis of *C*. *punctiferalis* larvae infected with *B*. *bassiana*. **(A)** Volcano plot. **(B)** GO enrichment analysis of DEPs. **(C)** KEGG pathway enrichment analysis of DEPs.

According to the differential protein screening criteria, a total of 3,431 proteins were identified, of which 197 were DEPs, 109 were up-regulated proteins, and 88 were down-regulated proteins (**Figure 2A**). In total, 117 DEPs were annotated to 132 GO terms on the basis of GO enrichment analysis. Among these, the most enriched GO terms were “aminoglycan metabolic process” (GO 0006022) in biological process and “serine-type peptidase activity” (GO 0008236) in molecular function (**Figure 2B**). According to the KEGG pathway enrichment analysis, 80 DEPs annotated to 150 KEGG maps. The most enriched KEGG pathways were “glutathione metabolism” (map 00480), “fatty acid metabolism” (map 01212), “ras signaling pathway” (map 04014), “epithelial cell signaling in *Helicobacter pylori* infection” (map 05120), and “PPAR signaling pathway” (map 03320) (**Figure 2C**).

### 3.3 Combined analysis of transcriptomic and proteomic

**Figure 3:**
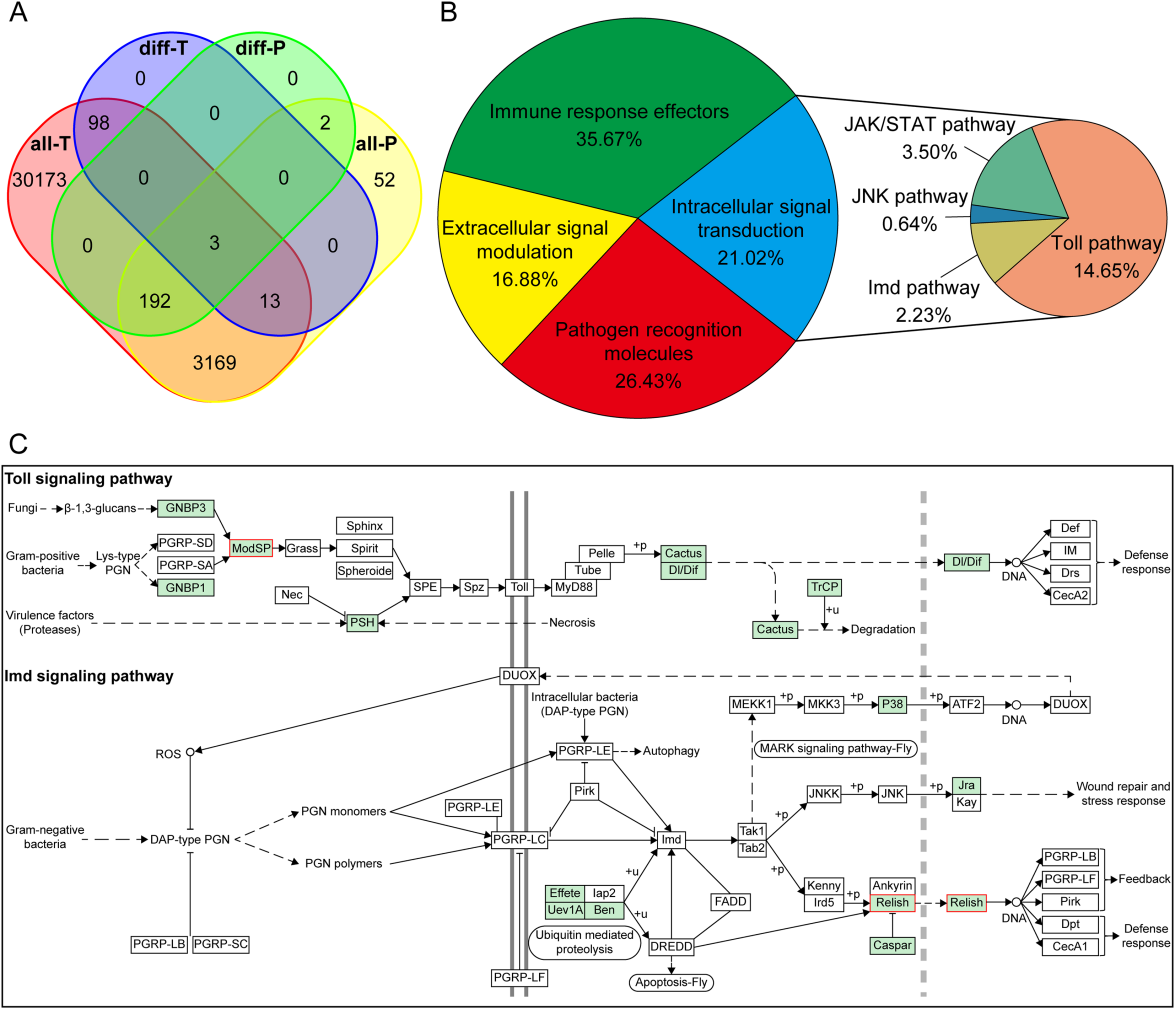
Integrated analyses of transcriptome and proteome data. **(A)** Venn diagram of transcriptome and proteome expression regulation. all-T, all genes obtained from the transcriptome; diff-T, DEGs were identified by the transcriptome analysis; all-P, all proteins obtained from the proteome; diff-P, DEPs were identified by the proteome analysis. **(B)** Distribution of immune-related genes and proteins from *C*. *punctiferalis* transcriptome and proteome. **(C)** The Toll and Imd signaling pathways. All identified proteins were indicated by green background boxes, and up-regulated differential proteins were marked by red boxes.

A scatter plot of transcriptome and proteome expression levels showed a Pearson correlation coefficient of 0.055, indicating a positive correlation between protein and corresponding gene expression (**Supplementary Figure 6**). The corresponding relationship between the transcriptome and proteome was shown in a Venn diagram through the integration of mRNA and protein information. Of the 3,431 identified proteins, 3,377 had corresponding transcripts in the transcriptome data. A total of 114 DEGs and 197 DEPs were identified, of which only three DEGs (DEPs) were screened together (**Figure 3A**).

In total, 314 immunity-related genes and proteins were identified from the transcriptome and proteome data. These genes and proteins were grouped according to their functions, including 83 pathogen recognition molecules, 53 extracellular signal modulation, 66 intracellular signal transduction, and 112 immune response effectors (**Figure 3B**).

The transcripts (proteins) with common significant differences were selected based on GO function and KEGG pathway enrichment analysis of the transcriptome and proteome. GO functional enrichment analysis included binding, structural constituent of cuticle, and catalytic activity. In KEGG pathway enrichment analysis, Toll and Imd signaling pathways (map 04624) and metabolic pathways (map 01100) were identified (**Supplementary Table 6**). In the Toll and Imd signaling pathways, a total of 15 proteins were identified in the KEGG pathway, of which two proteins were up-regulated differential proteins, namely ModSP (modular serine protease) and Relish (a nuclear factor kappa B, NF-*κ*B) (**Figure 3C**).

### 3.4 Overview and general features of CTLs

**Figure 4:**
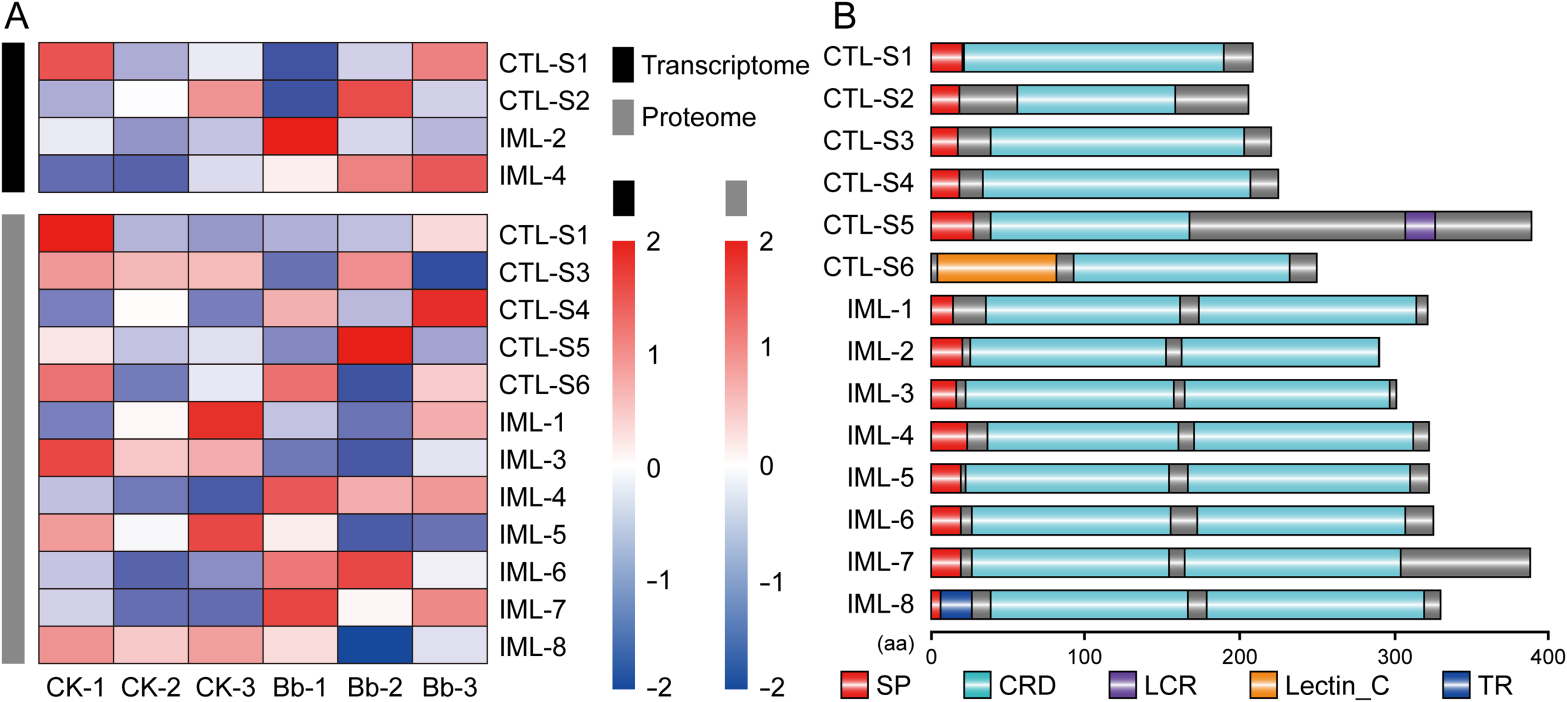
Schematic architectures and cluster heatmap of CTLs from *C*. *punctiferalis*. **(A)** Expression of CTLs from *C*. *punctiferalis* larvae based on the transcriptome and proteome data. CK-1, -2, and -3, larvae injected with sterile PBS; Bb-1, -2, and -3, larvae injected with *B*. *bassiana*. **(B)** Domain architectures of 14 CTLs. SP, signal peptide; CRD, carbohydrate recognition domain; LCR, low complexity region; TR, transmembrane region; aa, amino acid.

A total of 14 CTLs of *C*. *punctiferalis* were identified, four of which were found in the transcriptome and 12 in the proteome (**Figure 4A**). Based on CRD organization, six CTLs with a single CRD belonged to CTL-S, and the remaining eight CTLs with a dual CRD belonged to IMLs. CTL-X containing a single CRD and other functional domains were not found in the CTLs of *C*. *punctiferalis*. CTL-S1−S5 and IML-1−7 contained an N-terminal signal peptide, indicating the potential for secretion into the plasma. CTL-S6 was likely located in the cytoplasm due to the lack of N-terminal secretion signals. IML-8 contained a transmembrane region, suggesting a potential location on the cell membrane (**Figure 4B**). In total, 22 CRDs were identified from all CTLs, five of which contained the EPN (Glu-Pro-Asn) motif, six contained the QPD (Gln-Pro-Asp) motif, and 11 contained atypical motifs, such as EPD (Glu-Pro-Asp) (**Table 1**).

**Table 1:** Structural features of CTLs from *C*. *punctiferalis* larvae.

Name	ORF (aa)	MW (kDa)	pI	SP	CRD numbers	Motif
CTL-S1	207	23.17	5.47	1-21	1	APQ
CTL-S2	204	31.66	5.16	1-19	1	EPN
CTL-S3	219	25.34	6.65	1-18	1	QPD
CTL-S4	223	25.60	6.08	1-19	1	QPD
CTL-S5	385	45.38	10.36	1-28	1	VPE
CTL-S6	248	27.84	5.44	–	1	APQ
IML-1	319	35.84	5.65	1-15	2	DPN/EPN
IML-2	288	32.39	6.07	1-21	2	EPD/EPN
IML-3	299	34.54	4.95	1-17	2	SPN/QND
IML-4	320	35.32	5.84	1-24	2	EPD/QPD
IML-5	320	35.75	5.65	1-20	2	EPD/QPD
IML-6	322	37.05	4.92	1-20	2	EPN/QPD
IML-7	384	34.59	5.85	1-20	2	EPD/QPD
IML-8	327	36.98	5.70	1-21	2	EPD/EPN

The cluster heatmap showed that CTL-S1 and IML-4 were identified in both the transcriptome and proteome data. Notably, the expression of IML-4 (named *CpIML4*) was increased after *B*. *bassiana* infection compared to the control group. The expression of *CpIML4* exhibited significant up-regulation, especially in the proteome (**Figure 4A**). Subsequent trials were conducted to investigate this phenomenon.

### 3.5 Sequence analysis of *CpIML4*

**Figure 5:**
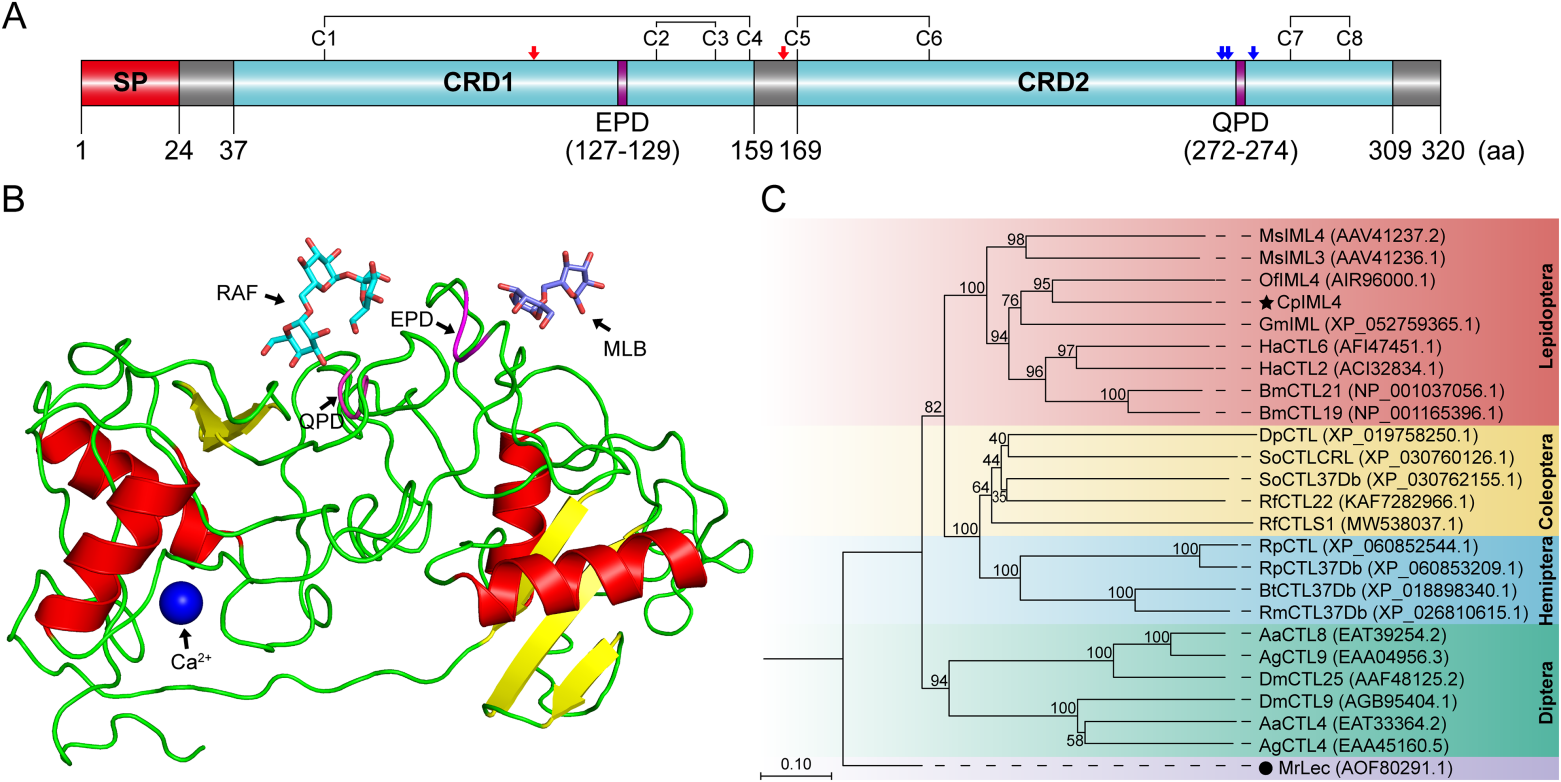
Sequence features and phylogenetic analysis of CpIML4. **(A)** The domain architecture of CpIML4. Eight highly conserved Cys residues generated four disulfide bonds (C1-C4, C2-C3, C5-C6 and C7-C8). Two N-glycosylation sites (red arrows) and three O-glycosylation sites (blue arrows) by glycosylation site analysis. SP, signal peptide; CRD1 and CRD2, carbohydrate recognition domain; EPD, Glu_127_-Pro_128_-Asp_129_; QPD, Gln_272_-Pro_273_-Asp_274_. **(B)** Three-dimensional structure model of CpIML4. RAF, raffinose; MLB, melibiose. **(C)** Phylogenetic analysis of CpIML4 and other insect CTLs. MrLec was used as out-group to root the phylogeny. Ms, *Manduca sexta*; Of, *Ostrinia furnacalis*; Cp, *Conogethes punctiferalis*; Gm, *Galleria mellonella*; Ha, *Helicoverpa armigera*; Bm, *Bombyx mori*; Dp, *Dendroctonus ponderosae*; So, *Sitophilus oryzae*; Rf, *Rhynchophorus ferrugineus*; Rp, *Rhopalosiphum padi*; Bt, *Bemisia tabaci*; Rm, *Rhopalosiphum maidis*; Aa, *Aedes aegypti*; Ag, *Anopheles gambiae*; Dm, *Drosophila melanogaster*; Mr, *Macrobrachium rosenbergii*.

The *C*. *punctiferalis* larval transcriptome data showed the full-length cDNA sequence of *CpIML4* was 987 bp. The ORF of *CpIML4* was 963 bp and a protein of 320 amino acids was encoded with a theoretical MW of 35.32 kDa and pI of 5.84. An N-terminal signal peptide (amino acid residues 1 to 24) and two conserved CRDs, CRD1 (amino acid residues 37 to 159) and CRD2 (amino acid residues 169 to 309), were predicted in CpIML4 (**Figure 5A**; **Supplementary Figure 7**). Ten conserved cysteine (Cys) residues within the amino acid sequence (Cys_58_, Cys_136_, Cys_150_ and Cys_158_ in CRD1, and Cys_169_, Cys_183_, Cys_200_, Cys_285_, Cys_299_, and Cys_308_ in CRD2) generated at least four disulfide bonds (Cys_58_-Cys_158_, Cys_136_-Cys_150_, Cys_169_-Cys_200_, and Cys_285_-Cys_299_) (**Figure 5A**). CpIML4 contained two motifs: EPD (Glu_127_-Pro_128_-Asp_129_) in CRD1 and QPD (Gln_272_-Pro_273_-Asp_274_) in CRD2 (**Figure 5A**; **Supplementary Figure 7**). The glycosylation site analysis showed that CpIML4 contained two N-glycosylation sites (Asn_107_ and Asn_166_) and three O-glycosylation sites (Ser_269_, Ser_270_, and Thr_276_) (**Figure 5A**; **Supplementary Figure 7**). Moreover, predictions made through TMHMM analysis suggested that CpIML4 may constitute an extracellular protein because of the lack of typical transmembrane domains (**Supplementary Figure 8**).

The 3D structure of CpIML4 contained four *α*-helices and five *β*-stands. CRD1 included one patchy *α*-helix, one *α*-helix, and two *β*-stands, whereas CRD2 included two *α*-helices and two *β*-stands. The Ca^2+^/sugar binding sites of the 3D structural model were predicted using COACH based on the I-TASSER server. There was a potential melibiose (MLB) site in CRD1, a potential Ca^2+^ site and a raffinose (RAF) site in CRD2. The MLB site contained amino acid residues (Glu_127_ and Asp_129_) in the EPD motif, and the galactose-type RAF site contained amino acid residues (Gln_272_ and Asp_274_) in the QPD motif (**Figure 5B**).

The amino acid sequence alignment demonstrated extensive coverage (≥ 89%) and high similarity between CpIML4 and the CTLs sequences of other insects. CpIML4 had the closest identity with OfIML4 (50.51%, AIR96000.1), followed by BmIML (42.61%, XP_004922068.1), HaCTL6 (40.68%, AFI47451.1), GmIML (40.34%, XP_052759365.1), SlIML (37.70%, XP_022827254.1), and MsIML (30.89%, XP_030038662.1). In CRD1, all insects contained an EPD motif, except for MsIML, which contained a typical EPN motif. In CRD2, CpIML4, OfIML4, and GmIML all contained a typical QPD motif, whereas BmIML, HaCTL6, and SlIML all contained a typical EPN motif. Ten conserved Cys residues were identified in CpIML4, and the disulfide bridges formed by these residues may affect the stability of the protein structure (**Supplementary Figure 9**).

A phylogenetic tree was constructed employing amino acid sequences from a total of 15 insect species. The CTL sequence of *M. rosenbergii* was used as an outgroup. CpIML4 was clustered in a subgroup of lepidopteran CTLs and shared a branch with OfIML4. The results indicated that CpIML4 and OfIML4 were the most homologous and performed similar functions (**Figure 5C**).

### 3.6 Temporal and spatial expression of *CpIML4*

**Figure 6:**
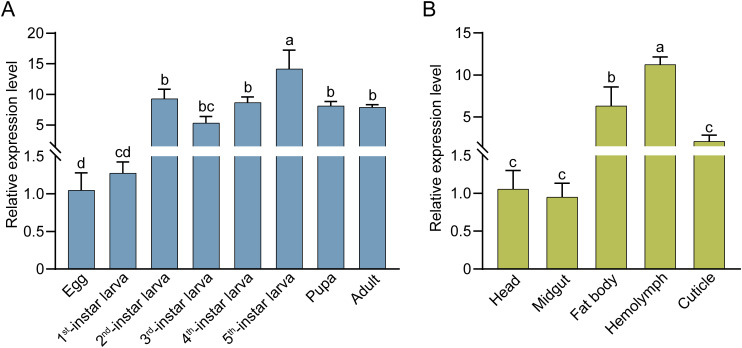
Expression profiles of CpIML4. **(A)** Relative expression levels of *CpIML4* at different developmental stages. **(B)** Relative expression levels of *CpIML4* in different tissues of 3-day-old 5^th^-instar larvae. The relative expression level is represented as mean ± SE. Different letters indicate significant differences by one-way ANOVA (*p* < 0.05).

The expression of *CpIML4* was detected throughout all the developmental stages (egg, larva, pupa, and adult), showing the lowest expression level in eggs and the highest expression level in 5^th^-instar larvae, followed by the other developmental stages. The relative expression level of *CpIML4* in 5^th^-instar larvae was 13.48 times higher than that in eggs (**Figure 6A**). Moreover, the expression levels of *CpIML4* exhibited variation in different larval tissues (head, midgut, fat body, hemolymph, and cuticle), with the highest expression level in the hemolymph, followed by the fat body, and lower expression levels in the midgut and head. The relative expression levels of *CpIML4* in the hemolymph and fat body were 11.85 and 6.66 times higher than those in the midgut and 10.66 and 5.99 times higher than those in the head, respectively (**Figure 6B**).

### 3.7 Effect of *CpIML4* RNAi on defense against fungal infection

**Figure 7:**
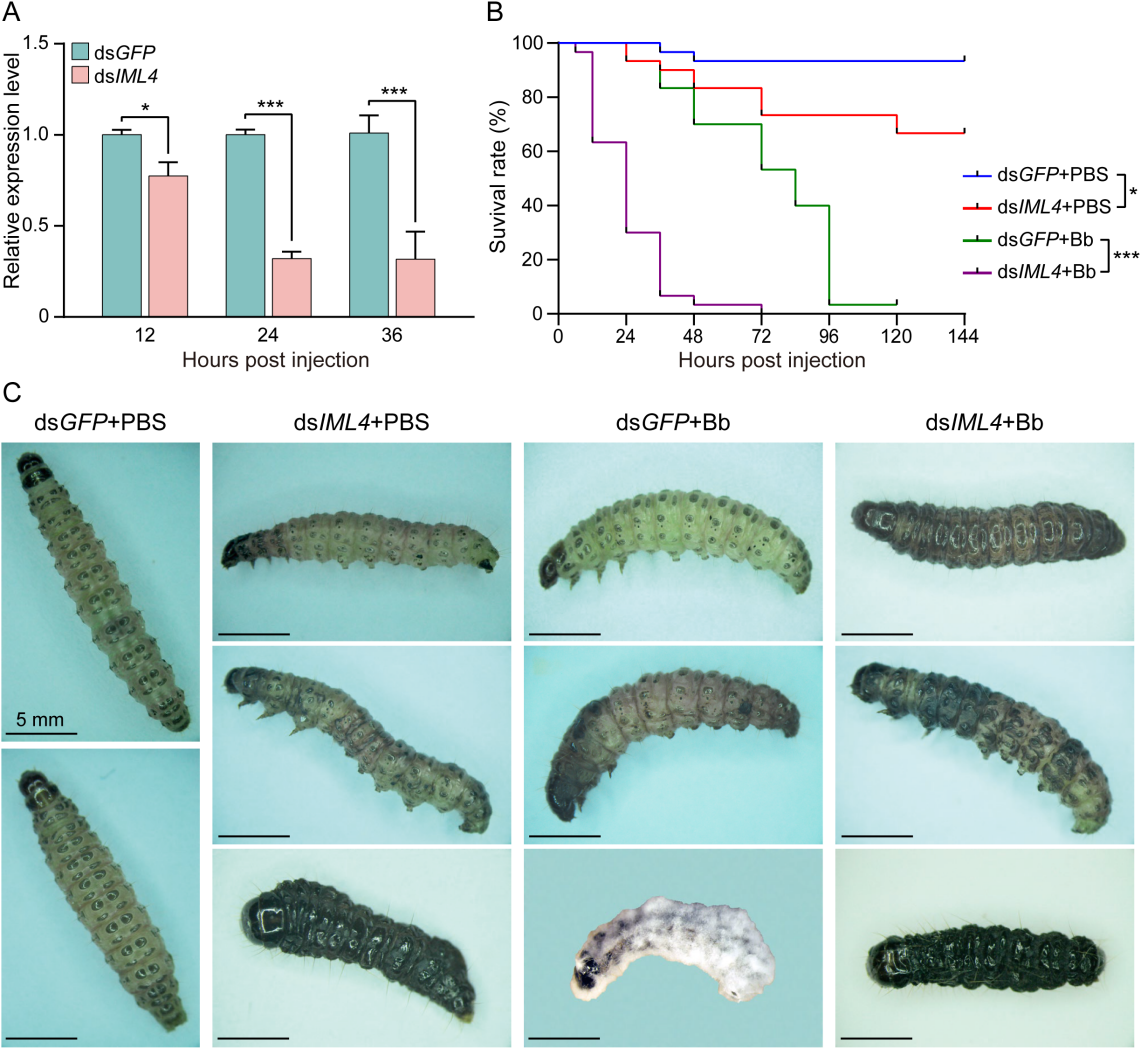
RNAi efficiency of *CpIML4* and effect of *CpIML4* RNAi on the survival and phenotype of *C*. *punctiferalis* larvae. **(A)** RNAi efficiency of *CpIML4*. **(B)** Survival rates of *C*. *punctiferalis* larvae infected with *B*. *bassiana* after *CpIML4* RNAi. **(C)** Pathological phenotypic changes of *C*. *punctiferalis* larvae infected with *B*. *bassiana* after *CpIML4* RNAi. Data are represented as mean ± SE. *, *p* < 0.05; **, *p* < 0.01; ***, *p* < 0.001. Scale bar, 5 mm.

Larvae were injected with ds*IML4* or ds*GFP* (control) and collected at different times to assess *CpIML4* RNAi efficiency. Compared with the control group, the expression levels of *CpIML4* were significantly reduced to 22.71%, 68.14%, and 68.43% at 12, 24, and 36 hpi, respectively (**Figure 7A**). The expression level of *CpIML4* was highly significant (*p* < 0.001) compared to the control group at 24 hpi, indicating that *CpIML4* RNAi was reliable for further study.

To explore the effect of *CpIML4* RNAi on larval susceptibility to fungal infection, the survival rates of *C*. *punctiferalis* larvae were recorded every 12 h. The results indicated that the survival rate of (ds*GFP*+PBS)-treated larvae was 93.33% at 144 h, and larvae underwent no significant changes and survived normally (**Figures 7B, C**). The survival rate of (ds*IML4*+PBS)-treated larvae was 66.67% at 144 h, which differed significantly (*p* < 0.05) from the control group (ds*IML4*+PBS) (**Figure 7B**). The cuticle of (ds*IML4*+PBS)-treated larvae showed black spots and developmental malformation, and the dead larvae turned black and wrinkled (**Figure 7C**). The survival rate of (ds*GFP*+Bb)-treated larvae was 3.33% at 120 h, and the time of death ranged between 72 to 96 hpi (**Figure 7B**). The cuticle of (ds*GFP*+Bb)-treated larvae appeared as black spots, and the dead larvae became dark red, mummified, and overgrown with hypha and conidia of *B*. *bassiana* (**Figure 7C**). All (ds*IML4*+Bb)-treated larvae died at 72 h, and the time of death was concentrated between 12 to 36 hpi. The survival rates differed significantly (*p* < 0.01) from that of the control group (ds*GFP*+Bb) (**Figure 7B**). The cuticle of the larval body exhibited a mass of black spots that later spread throughout the body, and the dead larvae turned black, dried, and wrinkled (**Figure 7C**). The results showed that *C*. *punctiferalis* larvae with a low expression of *CpIML4* were easily killed after *B*. *bassiana* infection and indicated that *CpIML4* critically influenced anti-fungal immunity.

## 4 Discussion

The severe damage caused by *C*. *punctiferalis* to maize has gradually attracted the interest of researchers in recent years in China. The findings of our long-term field investigation demonstrated that *C*. *punctiferalis* larvae exhibited powerful survival ability, even when exposed to severely destroyed and moldy maize (**Supplementary Figure 10**). This phenomenon is highly likely attributed to the insect’s well-developed innate immune systems, comprised of cellular and humoral immunity, to adapt to diverse environments (2). Cellular immune responses of *C*. *punctiferalis* against fungal infection have been proved in our previous results (38). This study further explores the immune mechanisms of *C*. *punctiferalis* in response to *B*. *bassiana* infection through the integrative transcriptomic and proteomic analyses.

Currently, multi-omics studies focus more on the integrative transcriptomic and proteomic analyses to reveal the relationship between mRNA levels and protein accumulation (15, 16, 43). However, the correlation between the transcriptome and proteome is not entirely consistent, a phenomenon has also been reported in other studies (44, 45). In this study, a total of 3,377 genes were found in both transcriptome and proteome data. A total of 114 DEGs and 197 DEPs were screened, in which only 3 DEGs (DEPs) were screened together. The number of DEGs (DEPs) was less than other lepidopteran insects, such as *H*. *armigera* and *Plutella xylostella* (46, 47). We speculated that the immune response of *C*. *punctiferalis* larvae was in the initial stage at 12 hpi, with the activation of some genes or proteins but no significant changes in expression levels. Moreover, two important immune signaling pathways, Toll and Imd signaling pathways, were identified through KEGG pathway enrichment analysis in the present study. The Toll and Imd signaling pathways are broadly conserved in insects apart from some hemipteran insects, such as *Acyrthosiphon pisum* and *B*. *tabaci*, which lack an intact Imd pathway (48). These two signaling pathways are important components of the innate immune system that can exist independently but cooperate with each other (49). The Toll signaling pathway regulates the expression of genes that encode antifungal and antibacterial peptides, whereas the Imd signaling pathway is activated by Gram-negative bacteria and regulates the expression of additional AMP genes (50, 51). Remarkably, the wounds caused by a microinjector and the integument injuries caused by a fungus can serve as entry points for bacterial infection, particularly Gram-negative bacteria (52, 53).

CTLs, a family of Ca^2+^-dependent carbohydrate-binding proteins, can be divided into three categories according to the CRD structure and phylogenetic relationships (CTL-S, IML, and CTL-X), and are distributed widely in insects (5, 14). For instance, *B*. *mori* has 11 CTL-Ss, seven IMLs, and five CTL-Xs (13); *M*. *sexta* includes 11 CTL-Ss, 17 IMLs, and five CTL-Xs (12); and *H*. *armigera* contains 11 CTL-Ss, 23 IMLs, and five CTL-Xs (11). In this study, 14 CTLs were identified in *C*. *punctiferalis* (including six CTL-Ss and eight IMLs) on the basis of transcriptome and proteome data; therefore, compared to these above-listed insects, fewer CTLs were identified in *C*. *punctiferalis*. The possible reason for this is that the number of CTLs may be species-specific and determined by factors such as the survival environment, selective pressure, and interaction with pathogenic microorganisms (14). However, it is highly possible that other unidentified transcripts may encode *C*. *punctiferalis* CTLs, and this deserves further detailed study. Interestingly, the quantitative distributions of CTLs in *C*. *punctiferalis* are highly similar to those of *O*. *furnacalis* CTLs (5 CTL-Ss and 9 IMLs) (10), and CTL-X is found in these two species. Nonetheless, *C*. *punctiferalis* is likely to possess CTL-X families, but additional sequence resources are required for further analysis and identification.

IMLs have mainly been found in lepidopteran insects, with small amounts also identified in coleopteran insects and crustaceans (12, 54). However, it is unclear whether they have a common ancestor or have arisen independently (12). In general, IMLs with two tandem CRDs show a broader spectrum of microorganisms and have a more extensive and stronger binding affinity to sugar ligands on the cuticle of pathogens than CTLs with a single CRD (12, 14). The binding specificity of CRDs depends on their canonical tripeptide motifs; for example, EPN and QPD are capable of binding to mannose- and galactose-type ligands, respectively (8, 9). Mutated motifs have been found in other insect IMLs, such as MsIML1 with QPR and EPN motifs in *M. sexta* (12), BmIML3 with EPD and EPS motifs in *B*. *mori* (13), and SeIML5 with EPN and QPN motifs in *Spodoptera exigua* (55). In this study, CpIML4 was found to possess two CRDs, of which CRD1 contained an EPD motif and CRD2 contained a canonical QPD motif. This finding is consistent with previous results obtained for OfIML4 in *O*. *furnacalis* (56). Based on its 3D structure, CpIML4 has a potential RAF site in CRD2 that can bind to galactose. In contrast, the EPD motif in CRD1 is a mutation in which Asn in the EPN motif is replaced with Asp, which can influence the binding affinity. Similar motifs have also been detected in OfIML4 of *O. furnacalis*; CRD2 with QPD may possess a galactose binding affinity, and CRD1 with EPD might lack mannose binding specificity (56). In *H*. *armigera* CTL, CRD1 with an EPD motif can instead bind to mannose and galactose, whereas CRD2 with an EPN motif possesses a broader carbohydrate binding spectrum than CRD1 (57). However, considering that only one amino acid residue is replaced, CpIML4 may nonetheless possess a potential binding specificity for mannose, galactose, or both to exert functions that recognize invading microbial pathogens. Present studies indicate that an amino acid sequence analysis alone does not accurately predict the binding specificity of CRDs (56, 57). Therefore, the detailed mechanisms underlying CpIML4 binding require further investigation.

An effective strategy using RNAi has recently been developed for insect pest management; this involves inhibiting the insect immune system, and the immune-related genes serve as potential RNAi targets for insect pest control (58). Insect CTLs are important PRRs that play critical roles in innate immunity (14). Some studies have shown that the knockdown of CTLs using RNAi can significantly increase the susceptibility of insects to pathogen infection. For example, knockdown of *MsIML2* markedly decreases the ability of *M. sexta* to withstand infection by *Photorhabdus* and reduces the hemolymph phenoloxidase activity of the insect (59). In addition, silencing *HaCTL11* or *HaCTL14* increases the rate of killing and reduced larval resistance to entomopathogenic fungi in *H*. *armigera* (60, 61), and the presence of *OfIML4* weakens the pathogenicity of *B. bassiana* in *O*. *furnacalis* (56). In this study, the knockdown of *CpIML4* led to larval death, and a significant mortality rate was seen compared to the control group. This suggests that *CpIML4* disrupts the normal growth and development of *C*. *punctiferalis* larvae and could serve as a potential target gene for RNAi-based control. Furthermore, combining RNAi with entomopathogenic fungi provides a promising strategy for insect pest management (62, 63). For instance, the silencing of *NlToll1* combined with fungal infection exhibits synergistic and highly effective insecticidal activity against *Nilaparvata lugens* (30). Compared to the control group (ds*IML4*+PBS), the survival rate of (ds*IML4*+Bb)-treated *C*. *punctiferalis* larvae was significantly decrease in the present study. We speculated that the knockdown of *CpIML4* of *C*. *punctiferalis* would lead to an obvious increase in susceptibility to *B*. *bassiana* infection and expedite the death of larvae. Notably, all larvae treated with (ds*IML4*+Bb) died, but no growth of *B*. *bassiana* was observed on the cadavers. Generally, *B*. *bassiana* requires a longer reaction time to kill the host insects and obtain sufficient nutrients to penetrate the cuticle and produce hyphae and conidia in this period. The larvae treated with (ds*IML4*+Bb) exhibited premature mortality and suffered excessive nutrient loss, which was insufficient for the optimal growth of *B*. *bassiana*. In other words, *B*. *bassiana* present in the larvae lacked the adequate time and nutrients to penetrate the insect cuticle. Given this, further studies could focus on combining RNAi with entomopathogenic fungi to explore potential synergistic or additive effects, thereby enhancing the efficacy of insect pest control.

In summary, integrative transcriptomic and proteomic analyses of *C*. *punctiferalis* in response to *B. bassiana* infection were performed using RNA-Seq and iTRAQ techniques. Based on the transcriptome and proteome data, the immune-related genes and proteins were screened, and a total of 14 CTLs were identified and characterized. Furthermore, *CpIML4* from *C*. *punctiferalis* was involved in the antifungal immune response. These findings provide a valuable resource for investigating the immune functions of CTLs from *C*. *punctiferalis*, and could contribute to the development of RNAi-based strategies for insect pest control.

## Data Availability

The datasets presented in this study can be found in online repositories. The names of the repository/repositories and accession number(s) can be found below: https://www.ncbi.nlm.nih.gov/, PRJNA1308180 http://www.proteomexchange.org/, PXD067498.
