# Supplementary material for: Transcriptomic and proteomic analyses of the immune responses of C-type lectin from Conogethes punctiferalis against fungal infection
Source: Front Immunol. 2026 Feb 26;17:1774776. doi: 10.3389/fimmu.2026.1774776 (PMC12979142; doi:10.3389/fimmu.2026.1774776)
Supplement: Supplementary file 1 [file DataSheet1.pdf]

Supplementary Material

1 Supplementary Tables

Table S1. The primers used in this study.

Name	Sequences (5'→3')
RP49-F	GCCATCAATCGGATCGCTATG
RP49-R	GCATGTGACGGGTCTTCTTG
IML4-F	ATGTTTAAATATACGAGTATTCTGTTGTCC
IML4-R	CTACATATTACTGTCGCTTATTATGACAGG
dsIML4-F	GAAGGATGGCTCAAGGTGTAC
dsIML4-R	TTCGTCGCAACTGGTCGTA
dsIML4-T7F	GAAGGATGGCTCAAGGTGTAC
dsIML4-T7R	TTCGTCGCAACTGGTCGTA
dsGFP-F	GCGACGTAAACGGCCACAAGT
dsGFP-R	GTACAGCTCGTCCATGCC
dsGFP-T7F	GGATCCTAATACGACTCACTATAGGGCGACGTAAACGGCCACAAGT
dsGFP-T7R	GGATCCTAATACGACTCACTATAGGGTACAGCTCGTCCATGCCGAG

Table S2. Summary of sequencing quality of *C. punctiferalis* larvae transcriptome.

Sample	Raw reads	Clean reads	Clean bases	Q30 (%)	GC content (%)
CpCK-1	20,433,014	19,676,707	5.90 G	93.33	49.65
CpCK-2	22,559,878	21,718,429	6.52 G	93.36	48.86
CpCK-3	23,008,005	22,045,869	6.61 G	93.67	47.76
CpBb-1	22,939,095	22,163,021	6.65 G	93.36	49.66
CpBb-2	22,235,461	21,392,053	6.42 G	93.50	49.62
CpBb-3	21,658,927	20,812,220	6.24 G	93.42	49.39

Table S3. Overview of RNA-Seq metrics from *C. punctiferalis* larvae transcriptome.

Type	Min length	Mean length	Max length	Total length	N50	Total nucleotides
Transcripts	301	1,501	31,398	90,568	2,295	135,925,102
Unigenes	301	1,365	31,398	33,648	2,247	45,921,295

Table S4. The annotation of unigenes from *C. punctiferalis* larvae transcriptome in database.

Annotated database	Number of unigenes	Percentage (%)
Annotated in NR	16,663	49.52
Annotated in NT	12,776	37.96
Annotated in KEGG	7,080	21.04
Annotated in SwissProt	10,880	32.33
Annotated in Pfam	11,980	35.60
Annotated in GO	11,978	35.59
Annotated in KOG	6,000	17.83
Annotated in all databases	3,203	9.51
Total unigenes	33,648	100

Table S5. Overview of iTRAQ metrics from *C. punctiferalis* larvae proteome.

Total spectra	Matched spectra	Peptide	Identified protein	All quantifiable protein
583,724	67,130	31,653	3,544	3,431

Table S6. Significant GO and KEGG pathway enrichment of DEPs with corresponding DEGs.

Gene ID	GO function	KEGG pathway	log_2FC (Protein)	*p* value (Protein)	log_2FC (Transcript)	*p* value (Transcript)
Cluster-4702.10273	binding	-	0.4091	0.014220	1.8122	0.012458
Cluster-4702.11231	structural constituent of cuticle	-	−0.3416	0.029681	−1.3428	0.016435
Cluster-4702.11487	binding	Toll and Imd signaling pathways	0.3217	0.042720	1.5007	0.003487
Cluster-4702.11794	binding	-	0.7994	0.004419	−2.1726	0.001418
Cluster-4702.11909	binding	-	0.2777	0.017495	1.5960	0.011718
Cluster-4702.12003	binding	-	−0.4775	0.010805	−6.3315	0.000007
Cluster-4702.16168	catalytic activity	metabolic pathways	0.2715	0.010986	−1.0990	0.004055
Cluster-7185.0	structural constituent of cuticle	-	−0.2666	0.044167	−3.5608	0.039931

2 Supplementary Figures

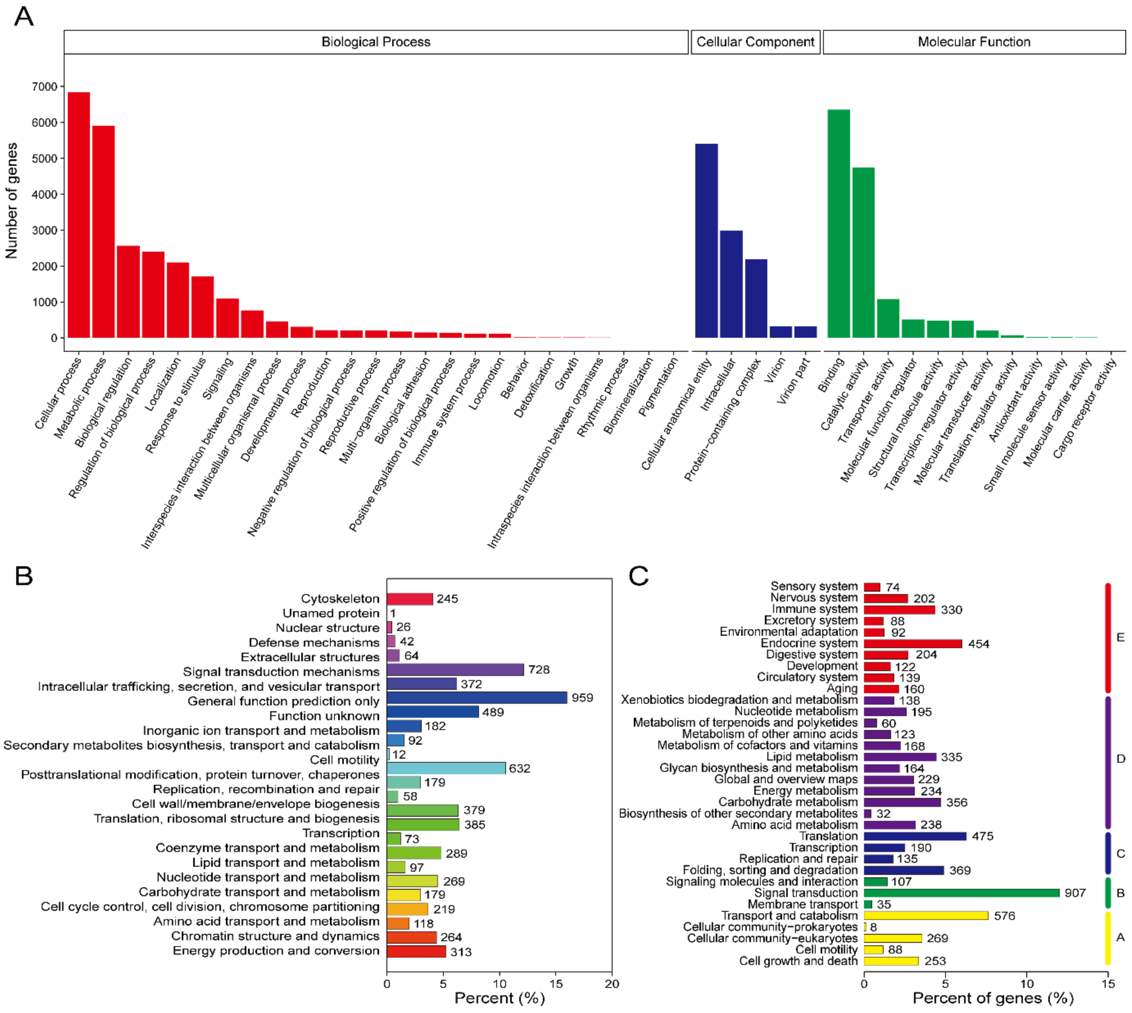

Figure S1. Functional annotation of *C. punctiferalis* larvae transcriptome. (A) GO functional annotation. (B) KOG functional annotation. (C) KEGG functional annotation.

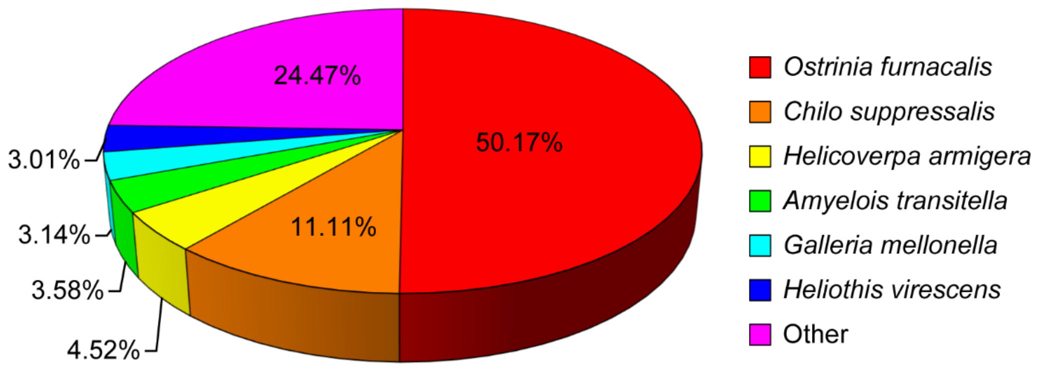

Figure S2. Species classification of the unigenes in NR database.

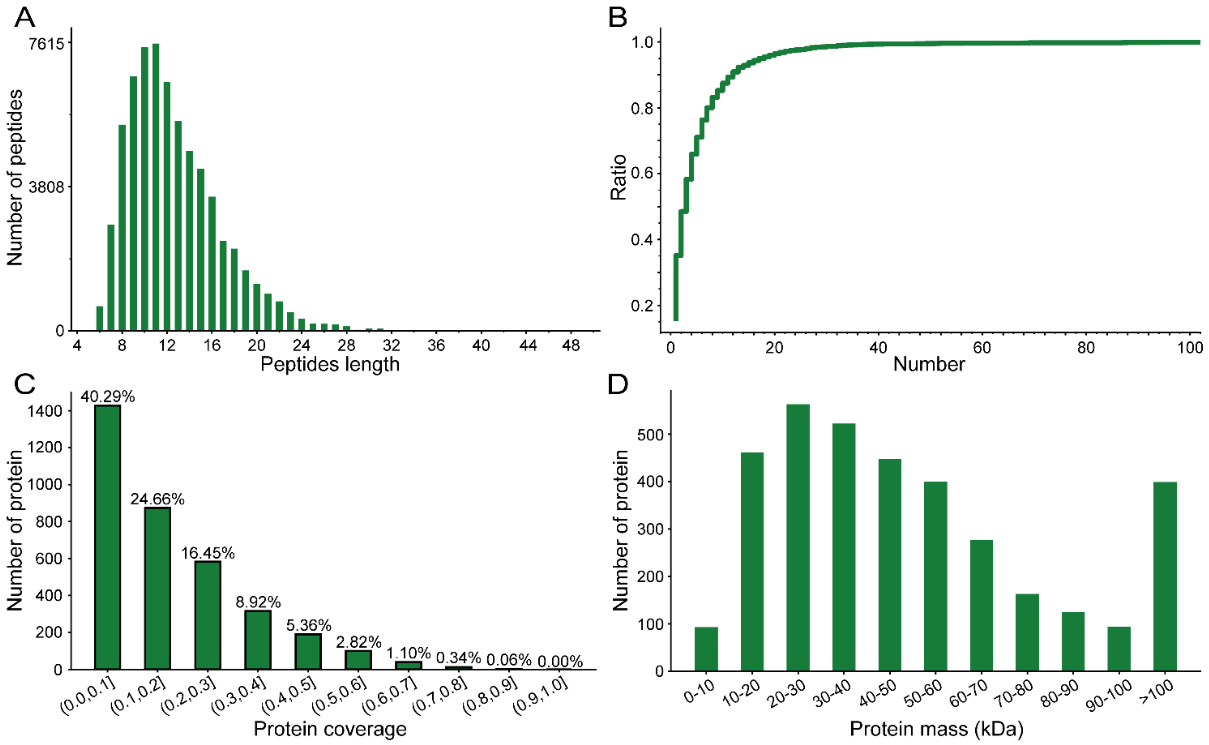

Figure S3. Quality control of *C. punctiferalis* larvae proteome. (A) peptide length distribution. (B) unique peptide number distribution. (C) protein coverage distribution. (D) protein molecular weight distribution.

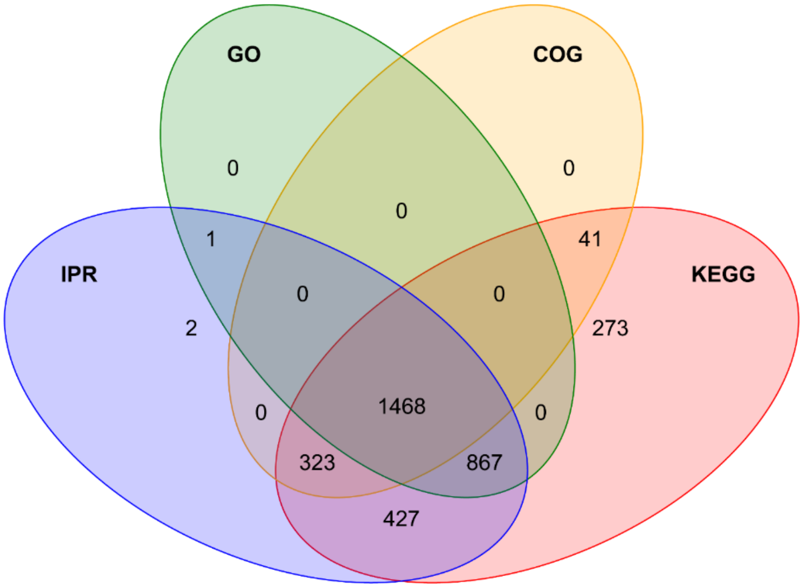

Figure S4. Venn diagram of functional annotation in GO, COG, KEGG, and IPR database.

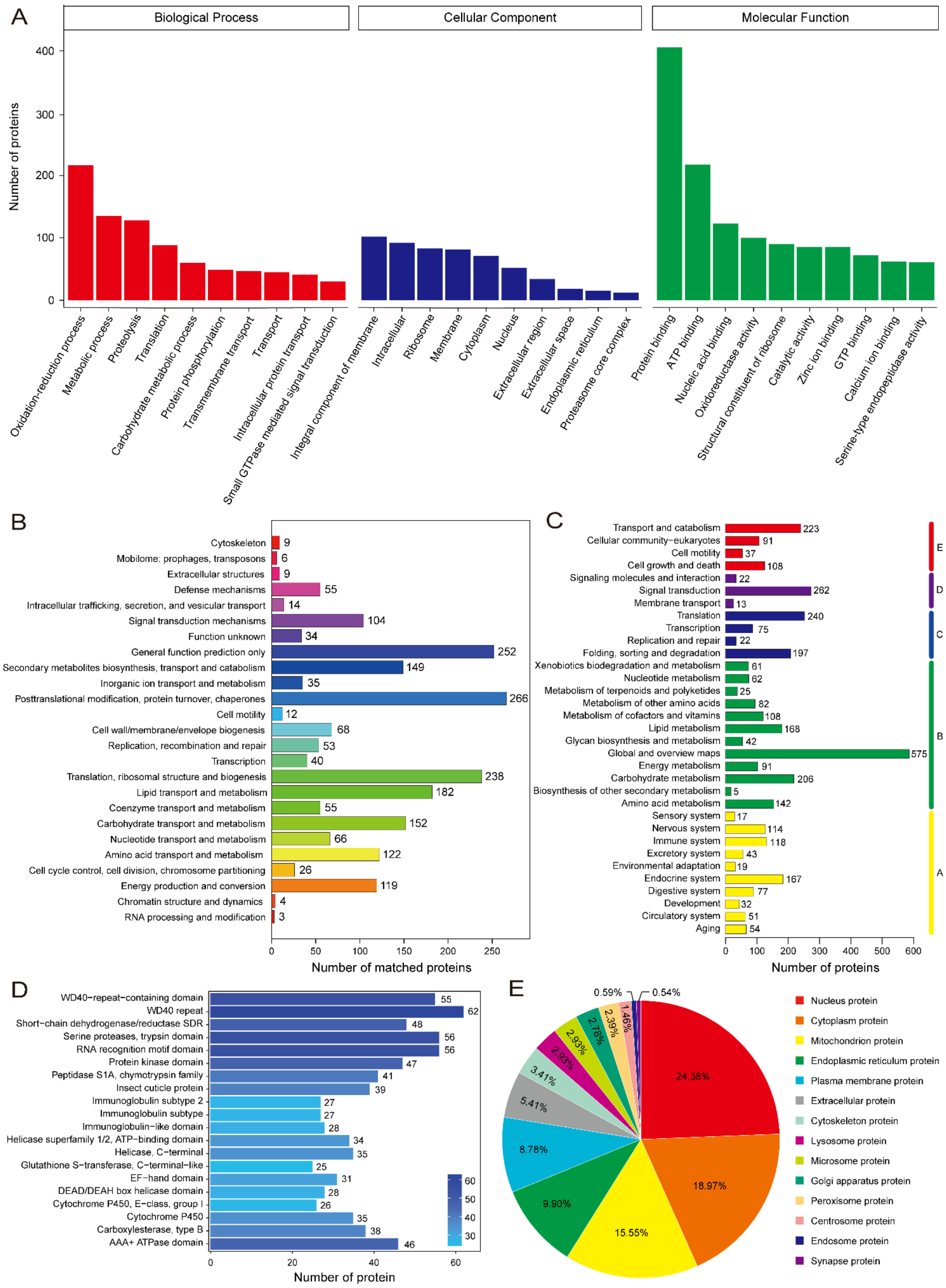

Figure S5. Functional annotation of *C. punctiferalis* larvae proteome. (A) GO functional annotation. (B) COG functional classification. (C) KEGG pathway annotation. (D) IPR protein domain annotation. (E) analysis of protein subcellular localization.

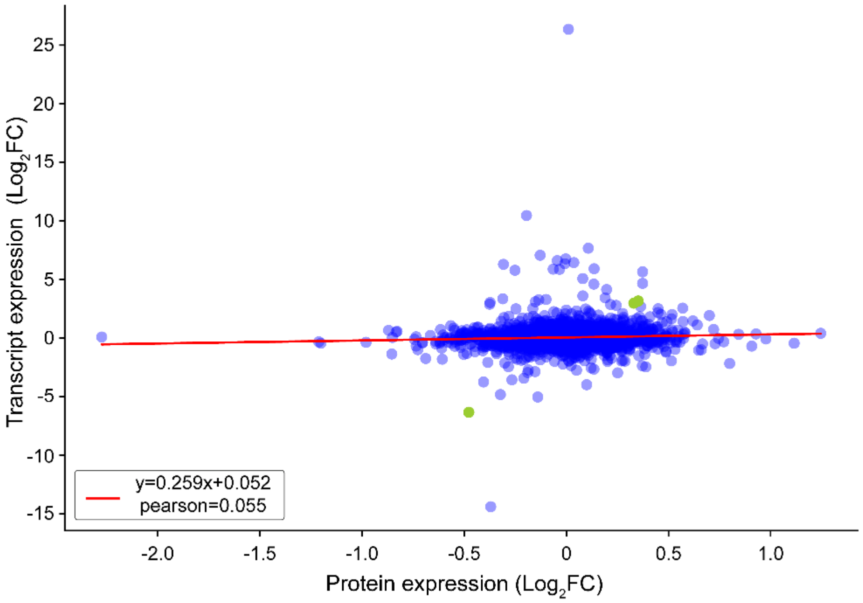

Figure S6. Correlation analysis of transcriptome and proteome expression. Green dots, DEPs; Blue dots, non-DEPs.

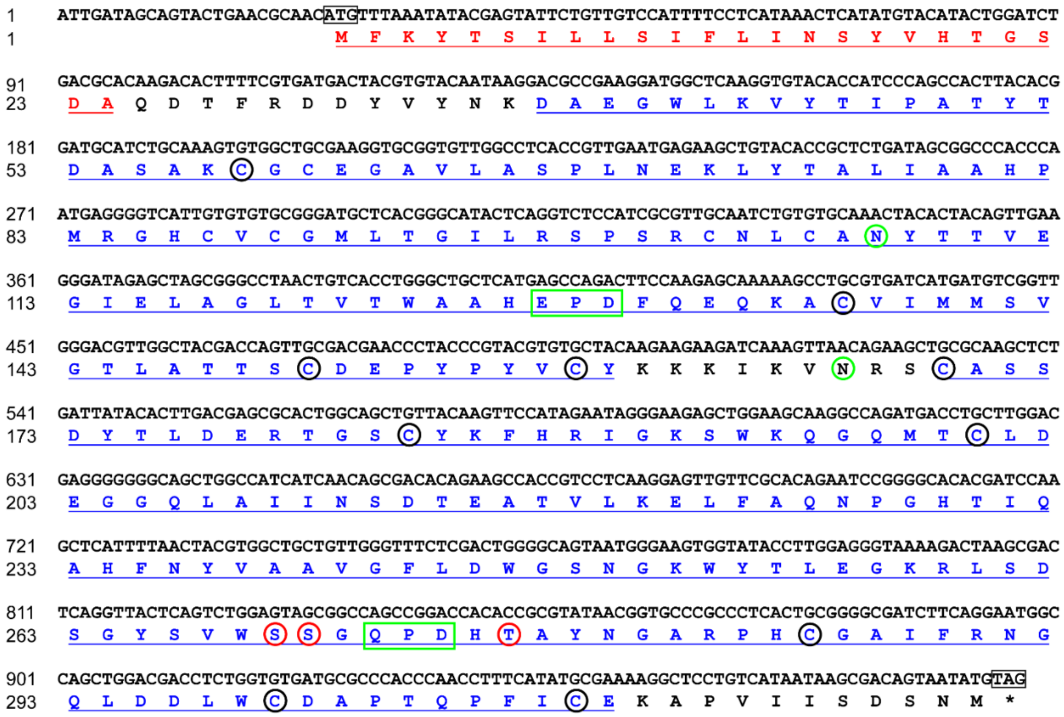

Figure S7. Nucleotide sequence of *CpIML4* ORF and the deduced amino acid sequence. The ATG and TAA codons are marked with black box, and the stop codon is shown as an asterisk. The predicted signal peptide and CRDs are underlined in red and blue, respectively. The EPD (Glu-Pro-Asn) and QPD (Gln-Pro-Asp) motifs are marked with green box. The cysteine (Cys) residues are marked with black circle. N-glycosylation sites and O-glycosylation sites are indicated by green and red circle, respectively.

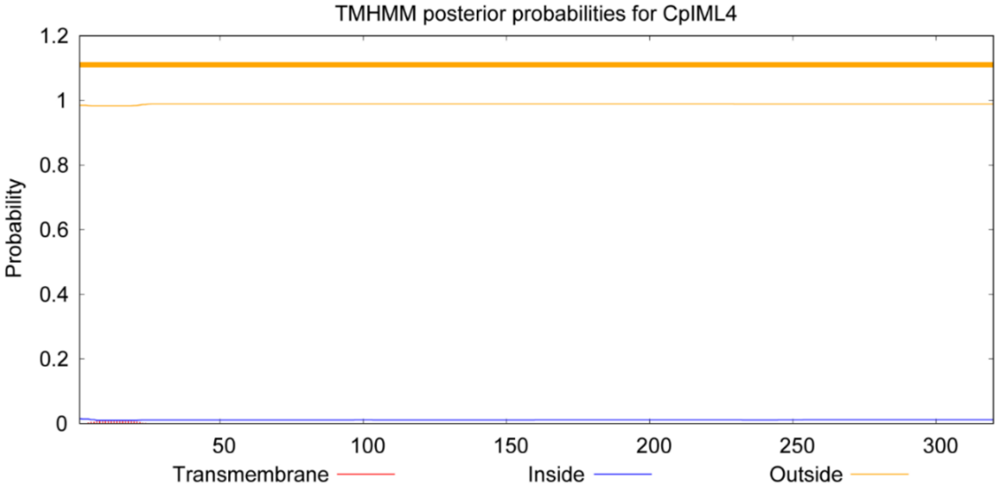

Figure S8. The result of transmembrane regions prediction. CpIML4 lacked typical transmembrane domains by TMHMM analysis.

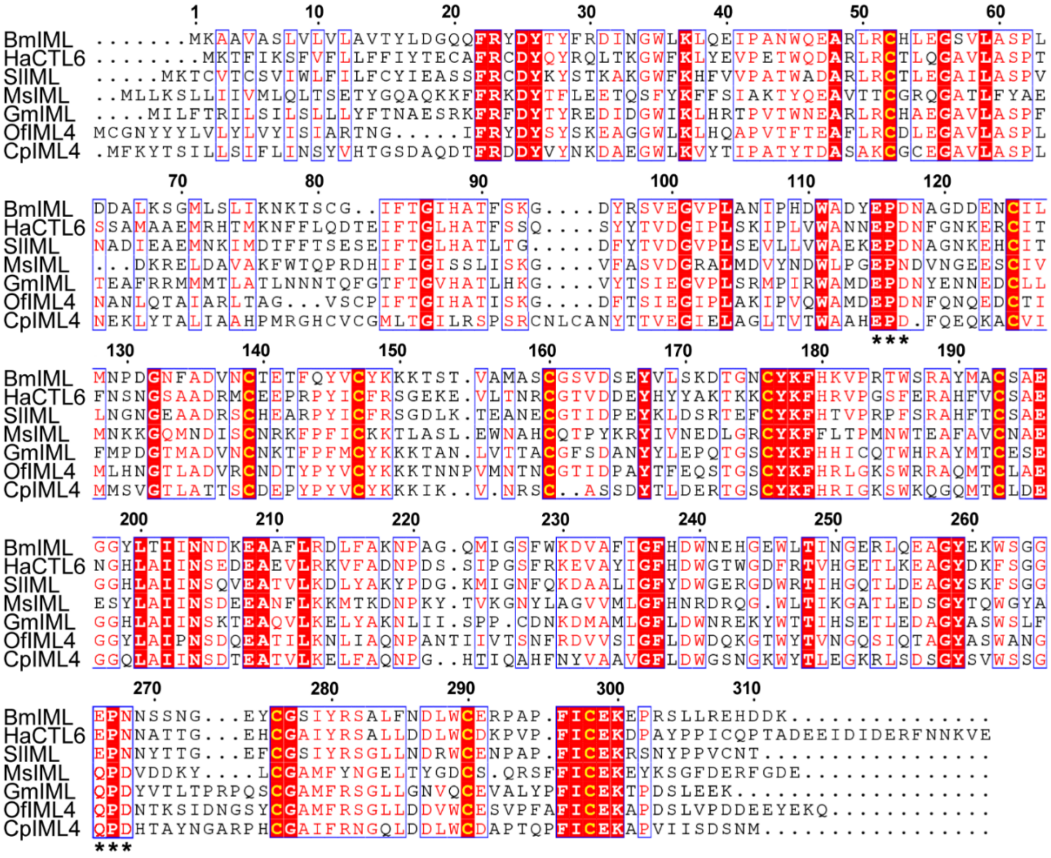

Figure S9. Multiple alignments of CpIML4 and other insects CTLs. Conserved Cys (C) residues are highlighted by yellow. The EPD, EPN, and QPD motifs are indicated by asterisks. BmIML, *Bombyx mori* IML (XP_004922068.1); HaCTL6, *Helicoverpa armigera* CTL6 (AFI47451.1); SlIML, *Spodoptera litura* (XP_022827254.1); MsIML, *Manduca sexta* IML (XP_030038662.1); GmIML, *Galleria mellonella* IML (XP_052759365.1); OfIML4, *Ostrinia furnacalis* IML4 (AIR96000.1); CpIML4, *Conogethes punctiferalis* IML4.

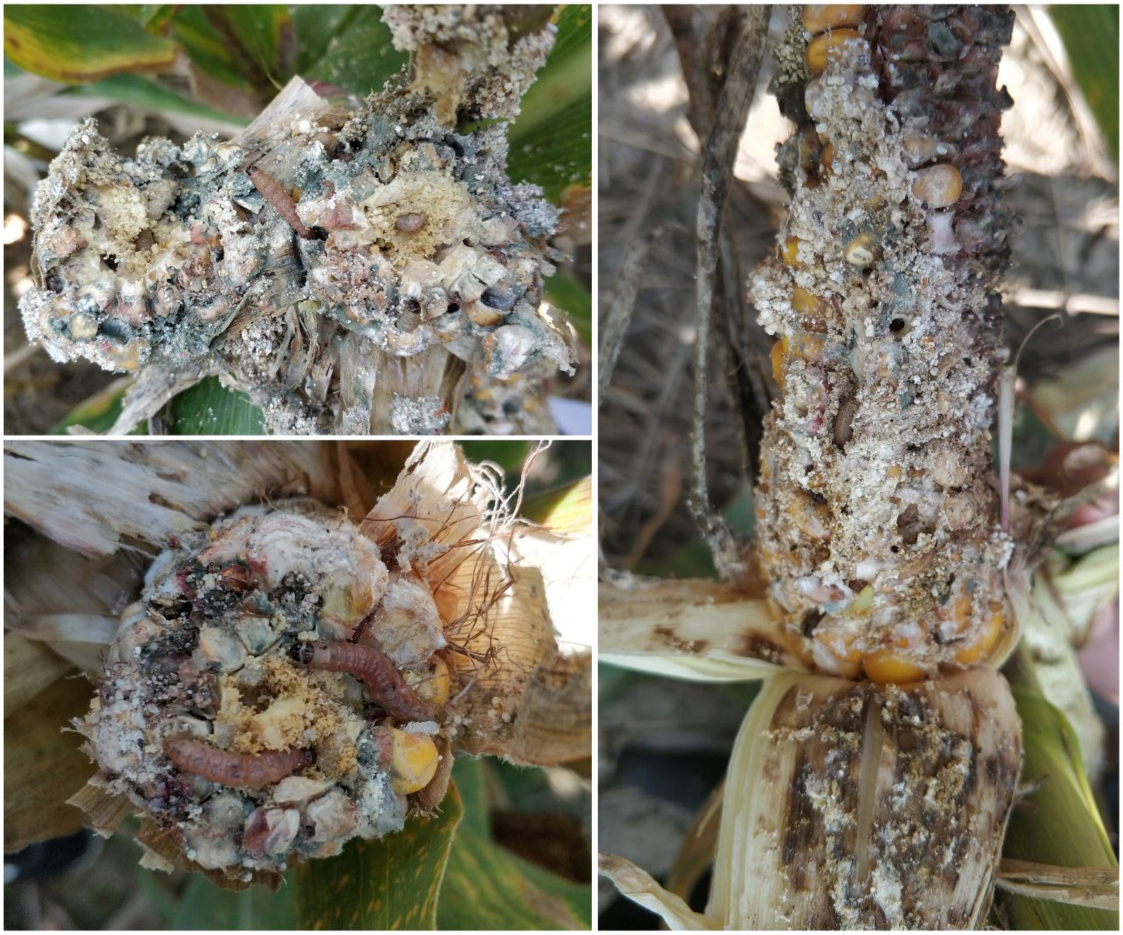

Figure S10. *C. punctiferalis* larvae on severely destroyed and moldy maize ears.
